# Inner approximation algorithm for generalized linear multiplicative programming problems

**DOI:** 10.1186/s13660-018-1947-9

**Published:** 2018-12-20

**Authors:** Yingfeng Zhao, Juanjuan Yang

**Affiliations:** School of Mathematical Science, Henan Institute of Science and Technology, Xinxiang, China

**Keywords:** Generalized multiplicative programming, Inner approximation algorithm, Geometric programming

## Abstract

An efficient inner approximation algorithm is presented for solving the generalized linear multiplicative programming problem with generalized linear multiplicative constraints. The problem is firstly converted into an equivalent generalized geometric programming problem, then some magnifying-shrinking skills and approximation strategies are used to convert the equivalent generalized geometric programming problem into a series of posynomial geometric programming problems that can be solved globally. Finally, we prove the convergence property and some practical application examples in optimal design domain, and arithmetic examples taken from recent literatures and GLOBALLib are carried out to validate the performance of the proposed algorithm.

## Introduction

In this paper, we focus on the following generalized linear multiplicative programming problem:

$$ (\mathrm{GLMP}):\quad \textstyle\begin{cases} \min &\phi _{0}(y)=\sum_{j=1}^{P_{0}}c_{0j}\prod_{t=1} ^{T_{0j}}(f_{0jt}(y))^{\gamma _{0jt}} \\ \textit{s.t.}&\phi _{i}(y)=\sum_{j=1}^{P_{i}}c_{ij}\prod_{t=1} ^{T_{ij}}(f_{ijt}(y))^{\gamma _{ijt}} \le 0,\quad i=1,2,\ldots,M, \\ &y \in Y^{0}=\{0< \underline{y}_{i}^{0}\le y_{i}\le \overline{y}_{i} ^{0}, i=1,2,\ldots,N\}, \end{cases} $$

where $c_{ij}$, $\gamma _{ijt}$, $i=0,1,\ldots,M$, $j=1,2,\ldots,p_{i}$, $t=1,2,\ldots,T_{ij}$ are all arbitrary real numbers; $p_{i}$, $T_{ij}$, $i=0,1,\ldots,M$, $j=1,2,\ldots,p_{i}$ are all positive integers and $f_{ijt}(y)$, $i=0,1,\ldots,M$, $j=1,2,\ldots,p_{i}$, $t=1,2,\ldots,T_{ij}$ are all affine functions defined on $R^{N}$ such that $f_{ijt}(y)>0$ for all $y\in Y^{0}$. Furthermore, we suppose that the interior of the feasible region for (GLMP) is not empty. Problem (GLMP) and its special cases are ubiquitous in optimal design applications, including power control, optimal doping profile, production planning, chemical equilibrium, heat exchanger network, digital circuit gate sizing, VLSI chip design, truss design, and so on [1–8]. And on the other hand, problem (GLMP) which corresponds to a nonlinear optimization problem with generalized linear multiplicative objective and constraint functions includes a large class of mathematical programs such as generalized geometric programming, multiplicative programming, sum of linear ratios problems, quadratic programming et al. [9–12]. Thus in this context, an algorithmic study of problem (GLMP) makes some theoretical and practical significance.

Algorithms for solving the special form of problem (GLMP) emerged endlessly. They are mainly classified as primal-based algorithms that directly solve the primal problem, dual-based algorithms that solve the dual problem, and adapted general nonlinear programming methods [13–15]. Recently, many works aimed at globally solving special forms of (GLMP) are presented, for example, global algorithms for signomial geometric programming problems, branch and bound algorithms for multiplicative programming with linear constraints, branch and reduction methods for quadratic programming problems, and sum of ratios problems are all in this category [16–21]. Despite these various contributions to their special forms, however, optimization algorithms for solving the general case of (GLMP) are still scarce. As far as we know, only [9] consider this general case, but only for (GLMP) with geometric constraints.

In this paper, we present an inner approximation algorithm for solving generalized linear multiplicative programming problem described as (GLMP). The (GLMP) is first converted into a generalized geometric programming problem, then the inner approximation algorithm relying on arithmetic-geometric mean inequality and magnifying-shrinking techniques is established. The algorithm works by solving a series of posynomial geometric programming problems. This strategy can be realized owing to the fact that recently developed solution methods can solve even large-scale posynomial geometric programming problems extremely efficiently and reliably [22]. The convergence property is proved and some examples taken from practical applications and recent literatures are performed to verify the efficiency of the presented algorithm. The experimental results show that the presented algorithm has a better capability to solve the (GLMP).

The remainder of this paper is organized in the following way. In Sect. 2, the equivalent generalized geometric programming problem is established and the inner approximation algorithm for solving (GLMP) is designed by utilizing arithmetic-geometric mean inequality and condensation techniques. The convergence property and error analysis of the algorithm are discussed in Sect. 3. Section 4 computationally investigates the performance of the inner approximation algorithm by solving some selective test examples. Some concluding remarks are proposed in the last section.

## Equivalent problem and algorithm development

In this section, the original problem (GLMP) is first transformed into an equivalent generalized geometric programming problem (EGGP) through variable substitution. And for convenience, problem (EGGP) will be further converted into generalized geometric programming with standard form described as formulation (Q). Then our focus will be shifted to solving the equivalent problem (Q). By utilizing the arithmetic-geometric mean inequality and condense techniques based on first order Taylor expansion, we can construct a posynomial geometric programming auxiliary problem (AQ) of the reformulated problem (Q) at each iterative point. Based on this, the proposed algorithm will be developed. The proposed algorithm works by solving a sequence of posynomial geometric programming problems.

### Equivalent problem

To solve the problem, we will first transform the (GLMP) into an equivalent problem (EGGP), where the objective and constraint functions are all generalized polynomial functions. To explain how such a reformulation is possible, we first compute $\underline{z}_{ijt}= \min_{y\in Y^{0}}f_{ijt}(y)$, $\overline{z}_{ijt}=\max_{y\in Y^{0}}f_{ijt}(y)$, then introduce some auxiliary variables $z_{ijt}$ such that $0<\underline{z}_{ijt} \le z_{ijt} \le \overline{z}_{ijt}$ for each $i=0,1,\ldots,M$, $j=1,2,\ldots,p_{i}$, $t=1,2,\ldots,T_{ij}$, and define vector *z* and an initial box $Z^{0}$ as follows:

$$\begin{aligned}& \begin{aligned} z={}&\{z_{011},z_{012}, \ldots,z_{01T_{01}},z_{021},z_{022},\ldots,z_{02T _{02}}, \ldots,z_{0p_{0}1},z_{0p_{0}2},\ldots,z_{0p_{0}T_{0p_{0}}}, \\ &{}z_{111},z_{112},\ldots,z_{11T_{11}},z_{121},z_{122}, \ldots,z_{12T _{12}},\ldots,z_{1p_{1}1},z_{1p_{1}2}, \ldots,z_{1p_{1}T_{1p_{1}}},\ldots, \\ &{}z_{M11},z_{M12},\ldots,z_{M1T_{M1}},z_{M21},z_{M22}, \ldots,z_{M2T _{M2}},\ldots,z_{Mp_{M}1},z_{Mp_{M}2},\ldots, \\ &{}z_{Mp_{M}T_{Mp_{M}}}\}\in R^{s}, \end{aligned} \\& Z^{0}=\bigl\{ z \in R^{s} \mid 0< \underline{z}_{ijt} \le z_{ijt} \le \overline{z}_{ijt}, i=0,1,\ldots,M, j=1,2,\ldots,p_{i}, t=1,2,\ldots,T_{ij}\bigr\} , \end{aligned}$$

where $s=\sum_{i=0}^{m}\sum_{j=1}^{p_{i}}T_{ij}$.

For convenience in exposition, we reintroduce some new notations as follows:

$$\begin{aligned}& T_{i}^{+}=\bigl\{ (j,t)\mid c_{ijt} \gamma _{ijt}>0, j=1,2,\ldots,p_{i}, t=1,2, \ldots,T_{ij}\bigr\} ,\quad i=0,1,2,\ldots,M, \\& T_{i}^{-}=\bigl\{ (j,t)\mid c_{ijt}\gamma _{ijt}< 0, j=1,2,\ldots,p_{i}, t=1,2,\ldots,T_{ij} \bigr\} ,\quad i=0,1,2,\ldots,M. \end{aligned}$$

With these new notations, problem (GLMP) can be further equivalently reformulated as the following problem:

$$ (\mathrm{EP}):\quad \textstyle\begin{cases} \min &\sum_{j=1}^{P_{0}}c_{0j}\prod_{t=1}^{T_{0j}}(z _{0jt})^{\gamma _{0jt}} \\ \textit{s.t.} &f_{ijt}(y)-z_{ijt}\le 0, \quad (j,t)\in T_{i}^{+},i=0,1,2,\ldots,M, \\ &z_{ijt}-f_{ijt}(y)\le 0, \quad (j,t)\in T_{i}^{-},i=0,1,2,\ldots,M, \\ &\sum_{j=1}^{P_{i}}c_{ij}\prod_{t=1}^{T_{ij}}(y_{ijt})^{ \gamma _{ijt}} \le 0,\quad i=1,2,\ldots,M, \\ &y \in Y^{0},\quad\quad z\in Z^{0}. \end{cases} $$

Upon the monotonicity of the function in problem (EP), it is not too hard to find that problems (GLMP) and (EP) have the same optimal solutions in the sense of the following theorem.

#### Theorem 1

$y^{*}$ *is an optimal solution for the* (GLMP) *if and only if* $(y^{*} ,z^{*})$ *is an optimal solution of* (EP), *where* $z^{*}_{ijt}=f_{ijt}(y^{*})$, $i=0,1,\ldots,M$, $j=1,2,\ldots,p_{i}$, $t=1,2,\ldots,T_{ip_{j}}$.

#### Proof

This theorem is quite easy to verify from the constructing process of problem (EP), thus the proof is omitted here. □

For convenience and without loss of generality, we can reformulate problem (EP) as the following generalized geometric programming problem (EGGP) by performing notation substitution.

$$ (\mathrm{EGGP}):\quad \textstyle\begin{cases} \min &\psi _{0}(x) \\ \textit{s.t.} & \psi _{i}(x) \le 0,\quad i=1,2,\ldots,m, \\ & x \in X^{0}, \end{cases} $$

where $x=(y,z)\in Y^{0}\times Z^{0}=X^{0}\subseteq R^{n}$, $n=N+s$, $m=M+s$, all of functions $\psi _{i}(x)$ have the generalized polynomial form, that is to say, it can be described as $\psi _{i}(x)=\sum_{t=1}^{r_{i}}\delta _{it}\prod_{j=1}^{n} (x_{j})^{ \theta _{itj}}$, and thus we only consider how to solve problem (EGGP) from now on.

### Implementable algorithm

In this part, we concentrate on how to design the inner approximation algorithm for solving the (EGGP). For this, we will perform some transformation and condensation strategies so that problem (EGGP) can be converted into a series of posynomial geometric programming problems which can be easily solved by using computer tools (such as CVX, GPLab).

To this end, we first denote all generalized polynomial functions in (EGGP) as

$$ \psi _{i}(x)=\psi _{i}^{+}(x)-\psi _{i}^{-}(x)\triangleq \sum_{j\in J_{i}^{+}} \delta _{ij}\prod_{j=1}^{n}(x_{j})^{ \theta _{ijt}}- \sum_{j\in J_{i}^{-}}\delta _{ij}\prod _{j=1}^{n}(x_{j})^{\theta _{ijt}}, $$

where

$$ J_{i}^{+}=\{j=1,2,\ldots,r_{i} \mid \delta _{ij}>0\},\quad\quad J_{i}^{-}=\{j=1,2,\ldots, r_{i} \mid \delta _{ij}< 0\}, \quad i=0,1,2,\ldots,m. $$

Note that the objective function can be rewritten as

$$ \psi _{0}(x)=\frac{\sum_{t=1}^{r_{0}}\delta _{0t}\prod_{j=1}^{n} (x_{j})^{\theta _{0tj}}}{\prod_{j=1}^{n} (x_{j})^{- \eta _{0j}}}=\frac{\sum_{t\in {T}_{0}^{+}}\delta _{0t}\prod_{j=1}^{n} (x_{j})^{\theta _{0tj}}}{\prod_{j=1}^{n} (x _{j})^{-\eta _{0j}}}+\frac{\sum_{t\in {T}_{0}^{-}}\delta _{0t} \prod_{j=1}^{n} (x_{j})^{\theta _{0tj}}}{\prod_{j=1}^{n} (x_{j})^{-\eta _{0j}}}, $$

where $\eta _{0j}=\min \{0,\theta _{0tj}\mid t=1,2,\ldots,r_{0}\}$, $j=1,2,\ldots,n$. If we denote

$$ \psi _{0}^{l}=\frac{\sum_{t\in {T}_{0}^{+}}\delta _{0t}\prod_{j=1}^{n} (\underline{x}^{0}_{j})^{\theta _{0tj}}}{\prod_{j=1}^{n} (\overline{x}^{0}_{j})^{-\eta _{0j}}}+\frac{\sum_{t\in {T}_{0}^{-}}\delta _{0t}\prod_{j=1}^{n} ( \overline{x}^{0}_{j})^{\theta _{0tj}}}{\prod_{j=1}^{n} ( \underline{x}^{0}_{j})^{-\eta _{0j}}} $$

and

$$ \psi _{0}^{u}=\frac{\sum_{t\in {T}_{0}^{+}}\delta _{0t}\prod_{j=1}^{n} (\overline{x}^{0}_{j})^{\theta _{0tj}}}{\prod_{j=1}^{n} (\underline{x}^{0}_{j})^{-\eta _{0j}}}+\frac{\sum_{t\in {T}_{0}^{-}}\delta _{0t}\prod_{j=1}^{n} ( \underline{x}^{0}_{j})^{\theta _{0tj}}}{\prod_{j=1}^{n} ( \overline{x}^{0}_{j})^{-\eta _{0j}}}, $$

then we have

$$ \psi _{0}^{l}\le \psi _{0}(x)\le \psi _{0}^{u}. $$

This will imply from $\psi _{0}^{l}$ that there exists a constant $\tau =-\psi _{0}^{l}+\epsilon $ with sufficiently small value $\epsilon >0$ such that $\psi _{0}(x)+\tau >0$, $\forall x \in X^{0}$. The reason for constructing the constant *τ* is that it will force the succedaneous objective function $\psi _{0}(x)+\tau >0$, and it is convenient for reformulating the following equivalent optimization problem:

$$ (\mathrm{Q}):\quad \textstyle\begin{cases} \min& x_{0} \\ \textit{s.t.} & \frac{\psi _{0}^{+}(x)+\tau }{\psi _{0}^{-}(x)+x_{0}} \le 1, \\ & \frac{\psi _{i}^{+}(x)}{\psi _{i}^{-}(x)} \le 1, \quad i=1,2,\ldots,m, \\ & x \in X^{0}, \quad\quad x_{0} \in [\psi _{0}^{l},\psi _{0}^{u}]. \end{cases} $$

In this representation, the objective function of problem (Q) is a positive linear function, and the constraints involve a special structure in the form of a ratio between two posynomials. Given that constraints in the form of a ratio between posynomials are not allowable in standard geometric programming [22], we attempt to approximate every posynomial denominator in constraints with monomial functions. This can be realized by utilizing the following arithmetic-geometric mean inequality:

$$ \varPhi (x)=\sum_{i=1}^{l}v_{i}(x) \ge \hat{\varPhi }(x)=\prod_{i=1}^{l} \biggl(\frac{v_{i}(x)}{\lambda _{i}(y)} \biggr)^{ \lambda _{i}(y)}, $$

where $v_{i}(x)$, $i=1,2,\ldots,l$, are monomial terms, and the parameter $\lambda _{i}(y)$ is obtained by computing $\lambda _{i}(y)=\frac{v _{i}(y)}{\varPhi (y)}$ so that $\hat{\varPhi }(x)$ is a best local monomial approximation of $\varPhi (x)$ near each fixed point *y* [22]. Based on this, the unallowable constraints of posynomial ratios form $\frac{\varPsi (x)}{\varPhi (x)}\le 1$ can be approximated with $\frac{ \varPsi (x)}{\hat{\varPhi }(x)}\le 1$. Applying this skill into all inapposite constraints of problem (Q), we can obtain the following auxiliary problem (AQ) which can be efficiently solved globally [22]:

$$ (\mathrm{AQ}): \quad \textstyle\begin{cases} \min &x_{0} \\ \textit{s.t.} & \frac{\psi _{0}^{+}(x)+\tau }{\tilde{\psi }_{0}(x,x_{0})} \le 1, \\ & \frac{\psi _{i}^{+}(x)}{\tilde{\psi }_{i}(x)} \le 1, \quad i=1,2,\ldots,m, \\ & x \in X^{0}, \quad\quad x_{0} \in [\psi _{0}^{l},\psi _{0}^{u}], \end{cases} $$

where $\tilde{\psi }_{0}(x,x_{0})$ equals $\psi _{0}^{-}(x)+x_{0}$ if $\psi _{0}^{-}(x)+x_{0}$ is monomial, and $\tilde{\psi }_{0}(x,x_{0})$ is the monomial approximation of $\psi _{0}^{-}(x)+x_{0}$ if $\psi _{0}^{-}(x)+x_{0}$ is posynomial; $\tilde{\psi }_{i}(x)$ equals $\psi _{i}^{-}(x)$ if $\psi _{i}^{-}(x)$ is monomial, and $\tilde{\psi }_{i}(x)$ is the monomial approximation of $\psi _{i}^{-}(x)$ if $\psi _{i}^{-}(x)$ is posynomial.

Based on the discussion above, now we can summarize the proposed algorithm for solving the (GLMP) as follows:

1. (Initialization) Reformulate the initial problem as the equivalent form described in problem (Q), then choose a feasible point $x^{(0)}$ and $x_{0}^{(0)}$ (if necessary) as the starting point, give out the solution accuracy $\vartheta \ge 0$, and set iteration counter $k:=0$.
2. (Inner approximation) At the $k_{th}$ iteration, replace each constraint with its inner approximation by computing the value of $\lambda _{i}(y)$ at $(x_{0}^{(k-1)},x^{(k-1)})$, if necessary.
3. (Posynomial condensation) Construct the auxiliary problem (AQ) and solve it to obtain $(x_{0}^{(k)},x^{(k)})$.
4. (Termination) If $\Vert x_{0}^{k}-x_{0}^{k-1} \Vert \le \vartheta $, then the algorithm can be terminated. Otherwise, set $k:=k+1$ and return to Step 2.

#### Remark 1

When performing the algorithm described above, one should choose a feasible interior point as the starting point. However, in the practical implementation, we often select an arbitrary point as the starting point when it is difficult to find a feasible interior point for some large-scale (GLMP) problems. This is mainly because the tool (GGPLab) we used for solving (AQ) can quickly produce a feasible interior point of problem (Q) [22].

## Convergence property analysis

In this section, we will briefly take into account the convergence properties of the above algorithm and evaluate the errors in objective and constraint functions produced by monomial approximation.

### Theorem 2

*The proposed algorithm either terminates within finite iterations with an KKT point for problem* (GLMP) *to be found*, *or the limit of any convergent sequence is a KKT point of the* (GLMP).

### Proof

First, according to the construction process of monomial approximation, we can easily verify that

1 $$ \frac{\psi _{0}^{+}(x)+\tau }{\psi _{0}^{-}(x)+x_{0}} \le \frac{\psi _{0}^{+}(x)+\tau }{\tilde{\psi }_{0}(x,x_{0})},\quad\quad \frac{\psi _{i}^{+}(x)}{ \psi _{i}^{-}(x)} \le \frac{\psi _{i}^{+}(x)}{\tilde{\psi }_{i}(x)}, \quad i=1,2,\ldots,m, $$

and

2 $$ \frac{\psi _{0}^{+}(x^{k})+\tau }{\psi _{0}^{-}(x^{k})+x_{0}^{k}} = \frac{ \psi _{0}^{+}(x^{k})+\tau }{\tilde{\psi }_{0}(x^{k},x_{0}^{k})}, \quad\quad \frac{ \psi _{i}^{+}(x^{k})}{\psi _{i}^{-}(x^{k})} = \frac{\psi _{i}^{+}(x^{k})}{ \tilde{\psi }_{i}(x^{k})}, \quad i=1,2,\ldots,m. $$

Second, we can also prove that

3 $$ \begin{gathered} \bigtriangledown \biggl(\frac{\psi _{0}^{+}(x^{k})+\tau }{\psi _{0}^{-}(x ^{k})+x_{0}^{k}} \biggr) = \bigtriangledown \biggl(\frac{\psi _{0}^{+}(x ^{k})+\tau }{\tilde{\psi }_{0}(x^{k},x_{0}^{k})} \biggr), \\ \bigtriangledown \biggl(\frac{\psi _{i}^{+}(x^{k})}{\psi _{i}^{-}(x^{k})} \biggr) = \bigtriangledown \biggl( \frac{\psi _{i}^{+}(x^{k})}{\tilde{\psi } _{i}(x^{k})} \biggr), \quad i=1,2,\ldots,m. \end{gathered} $$

Finally, we know the interior of the feasible region is not empty and all constraints in problem (AQ) are geometric-convex. This will suggest that the feasible region of problem (AQ) satisfies Slater’s constraint qualification condition. Thus based on (1)–(3) and according to Theorem 1 in [23], we conclude that the sequent solutions of problem (AQ) converge to the KKT point for problem (Q), thus for problem (GLMP). □

### Remark 2

Although the above algorithm can only obtain a KKT point for problem (Q), according to the special structure of the objective function of problem (Q) and the distinctive characteristics described in [23], we find that the KKT point found by the proposed algorithm is always a global optimal solution for problem (Q).

### Remark 3

Suppose $(x^{*}, x_{0}^{*})$ is the final solution obtained by the presented algorithm, we can evaluate the errors in objective and constraint functions produced by monomial approximation by the following formulas:

$$\begin{aligned}& \begin{aligned} \varTheta _{0} & = \bigl\vert \bigl(\psi _{0}^{+}\bigl(x^{*}\bigr)+\tau -\psi _{0}^{-}\bigl(x ^{*}\bigr)-x^{*}_{0} \bigr) - \bigl(\psi _{0}^{+}\bigl(x^{*}\bigr)+ \tau - \tilde{\psi }_{0}\bigl(x^{*},x^{*}_{0} \bigr) \bigr) \bigr\vert \\ & = \bigl\vert \psi _{0}^{-}\bigl(x^{*} \bigr)+x^{*}_{0}-\tilde{\psi }_{0} \bigl(x^{*},x^{*} _{0}\bigr) \bigr\vert , \end{aligned} \\& \begin{aligned} \varTheta _{i} & = \bigl\vert \bigl(\psi _{i}^{+}\bigl(x^{*}\bigr)-\psi _{i}^{-}\bigl(x^{*}\bigr) \bigr) - \bigl(\psi _{i}^{+}\bigl(x^{*}\bigr)-\tilde{\psi }_{i}\bigl(x^{*}\bigr) \bigr) \bigr\vert \\ & = \bigl\vert \psi _{i}^{-}\bigl(x^{*} \bigr)-\tilde{\psi }_{i}\bigl(x^{*}\bigr) \bigr\vert , \quad i=1,2,\ldots,m. \end{aligned} \end{aligned}$$

## Computational experiments

To test the proposed algorithm in terms of efficiency and solution quality, we performed some computational examples on a personal computer with Intel Xeon(R) CPU 2.40 Ghz and 4 GB memory. The code base is written in matlab 2014a and interfaces GGPLab for the standard geometric programming problems.

We consider some instances of problem (MIQQP) from some recent literature [9, 24–27] and MINLPLib [28]. Among them, Examples 1, 3, and 4 are three practical applications of (GLMP). Examples 2, 5, 6, 7, 8, and 9 are taken from recent literature for comparison analysis. Example 10 is an example for testing the influence of the numerical experiments for different initial points. Examples 11–13 are three examples from GLOBALLib [29], a collection of nonlinear programming models. The last example is a generalized linear multiplicative programming problem with randomized objective and constraint functions.

### Example 1

(see [24])

$$ \textstyle\begin{cases} \min &x_{1}+{x_{2}}+x_{3} \\ \text{s.t.} & 833.33252x_{1}^{-1}x_{4}x_{6}^{-1} +100x_{6}^{-1} \le 1, \\ & 1250x_{2}^{-1}x_{5}x_{7}^{-1} +x_{4}x_{7}^{-1}-1250x_{2}^{-1}x_{4}x _{7}^{-1} \le 1, \\ & 1{,}250{,}000x_{3}^{-1}x_{8}^{-1} +x_{5}x_{8}^{-1}-2500x_{3}^{-1}x_{5}x _{8}^{-1} \le 1, \\ & 0.0025x_{4} +0.0025x_{6} \le 1, \\ & -0.0025x_{4} +0.0025x_{5} +0.0025x_{7}\le 1, \\ & 0.01x_{8}-0.01x_{5} \le 1, \\ & 100\le x_{1}\le 10{,}000, \\ & 1000\le x_{2},x_{3}\le 10{,}000, \\ & 10\le x_{i}\le 1000,\quad i=4,5,\ldots,8. \end{cases} $$

This special instance of (GLMP) is first proposed to deal with the optimal design of heat exchanger networks [30]. When performing the algorithm for solving this instance, we choose $(500, 500, 4200, 500, 400, 340, 300, 600)$ as the starting point, the termination error was set to be $\vartheta =1\times 10^{-6}$. The proposed algorithm terminates after 3.74 seconds (CPU time) with solution $(579.326059, 1359.9445, 5109.977472, 182.019317, 295.600901, 217.980682, 286.418416, 395.600901)$ and optimal value 6944.248031 to be found, and the number of iterations is 21. While the method of Tsai and Lin [24] takes nearly one hour and forty minutes for solving this example, and they obtain a solution $(578.973143, 1359.572730, 5110.701048, 181.9898, 295.5719,218.0101, 286.4179, 395.5719)$ with the optimal value 7049.24682.

### Example 2

(see [9])

$$ \textstyle\begin{cases} \min &(x_{1}+{x_{2}}+1)^{1.1}(x_{1}+{x_{2}}+2)^{-1.1}(x_{1}+{x_{2}}+3)^{1.2}(x _{1}+{x_{2}}+4)^{-1.2} \\ &{}-(x_{1}+{x_{2}}+6)^{1.1}(x_{1}+{x_{2}}+5)^{-1.1}(x_{1}+{x_{2}}+8)^{1.2}(x _{1}+{x_{2}}+7)^{-1.2} \\ \text{s.t.} &x_{1}^{-1}x_{2}^{0.5} +x_{1}x_{2} \le 4, \\ & 1\le x_{1} \le 2,\quad\quad 1\le x_{2} \le 2. \end{cases} $$

In this example, both the objective function and the constraint function are generalized linear multiplicative functions. This example is taken from Jiao, Liu, and Zhao [9]. For solving this problem with the branch and bound algorithm, quite a lot of CPU times need to be consumed; however, we only expend less than two seconds for solving it to global optimality. In the iteration process, we select $(1.5,1.5)$ as the starting point, the termination error was also set to be $\vartheta =1\times 10^{-6}$.

### Example 3

(see [25])

$$ \textstyle\begin{cases} \min &0.5(x_{1}-10)x_{2}^{-1}-x_{1} \\ \text{s.t.} &x_{2}x_{3}^{-1}+x_{1}+0.5x_{1}x_{3}\le 100, \\ &1\le x_{i}\le 100,\quad i=1,2,3. \end{cases} $$

This example is a signomial geometric programming problem (special case of (GLMP)) which is used to optimize the design of a membrane separation process [25]. Lin and Tsai solved it with a range reduction method and obtained an optimal solution with optimal value −83.249728. For obtaining this solution, the range reduction method spend about 22 second (CPU time). Here, our algorithm terminated after 11 iterations and obtained the optimal solution $(87.614446,8.754375,1.413643,19.311410)$ with optimal value −85.68859, the algorithm implementation took about 0.942 seconds. In the algorithm implementation, we choose the initial upper bound $(100,100,100)$ as the starting point, the termination error was set to be $\vartheta =1\times 10^{-6}$.

### Example 4

(see [24])

$$ \textstyle\begin{cases} \min &-x_{1}+0.4x_{1}^{0.67}x_{7}^{-0.67}-x_{2}+0.4x_{2}^{0.67}x_{8} ^{-0.67}+10 \\ \text{s.t.} &0.0588x_{5}x_{7}+0.1x_{1} \le 1, \\ &4x_{3}x_{5}^{-1}+2x_{3}^{-0.71}x_{5}^{-1}+0.0588x_{3}^{-1.3}x_{7} \le 1, \\ &0.0558x_{6}x_{8}+0.1x_{1}+0.1x_{2} \le 1, \\ &4x_{4}x_{6}^{-1}+2x_{4}^{-0.71}x_{6}^{-1}+0.0588x_{4}^{-1.3}x_{8} \le 1, \\ &0.1\le x_{i} \le 10,\quad i=1,2,\ldots,8. \end{cases} $$

This example is a mathematical model born from optimal design of a reactor. For solving it, we select $(7,7,7,7,7,7,7,7)$ as the starting point, the termination error was set to be $\vartheta =1\times 10^{-6}$. The proposed algorithm terminates after 7.123 seconds (CPU time) with solution $(6.350802, 2.365111, 0.670723, 0.597563, 5.951950, 5.537204,1.042703,0.415594)$ and optimal value 3.908619 to be found, and the number of iterations is 44. While Tsai and Lin [24] spent nearly 56 minutes and 312 seconds for solving this example and obtained a solution $(6.473164, 2.238234, 0.664955, 0.591012, 5.930263,5.523595,1.011611,0.397171)$ with the optimal value 3.95109.

### Example 5

(see [9])

$$ \textstyle\begin{cases} \min &3.7x_{1}^{0.85}+1.985x_{1}+700.3x_{2}^{-0.75} \\ \text{s.t.} &0.7673x_{2}^{0.05}-0.05x_{1} \le 1, \\ &0.1\le x_{1} \le 5,\quad\quad 380\le x_{2} \le 450. \end{cases} $$

### Example 6

(see [9])

$$ \textstyle\begin{cases} \min &-x_{1}+0.4x_{1}^{0.67}x_{3}^{0.67} \\ \text{s.t.} &0.05882x_{3}x_{4}+0.1x_{1} \le 1, \\ &4x_{2}x_{4}^{-1}+2x_{2}^{-0.71}x_{4}^{-1}+0.05882x_{2}^{-1.3}x_{3} \le 1, \\ &0.1\le x_{i} \le 10,\quad i=1,2,3,4. \end{cases} $$

### Example 7

(see [27])

$$ \textstyle\begin{cases} \min & 5.3578x_{3}^{2}+0.8357{{x}_{1}}{{x}_{5}}+37.2392{{x}_{1}} \\ \textit{s.t.} & 0.00002584{{x}_{3}}{{x}_{5}}-0.00006663{{x}_{2}}{{x}_{5}}-0.0000734 {{x}_{1}}{{x}_{4}}\le 1, \\ & 0.000853007{{x}_{2}}{{x}_{5}}+0.00009395{{x}_{1}}{{x}_{4}}-0.00033085 {{x}_{3}}{{x}_{5}}\le 1, \\ & 1330.3294x_{2}^{-1}x_{5}^{-1}-0.42{{x}_{1}}x_{5}^{-1}-0.30586x_{2} ^{-1}x_{3}^{2}x_{5}^{-1}\le 1, \\ & 0.00024186{{x}_{2}}{{x}_{5}}+0.00010159{{x}_{1}}{{x}_{2}}+0.00007379x _{3}^{2}\le 1, \\ & 2275.1327x_{3}^{-1}x_{5}^{-1}-0.2668{{x}_{1}}x_{5}^{-1}-0.40584 {{x}_{4}}x_{5}^{-1}\le 1, \\ & 0.00029955{{x}_{3}}{{x}_{5}}+0.00007992{{x}_{1}}{{x}_{3}}+0.00012157 {{x}_{3}}{{x}_{4}}\le 1, \\ & 78\le {{x}_{1}}\le 102,\quad\quad 33\le {{x}_{2}}\le 45,\quad\quad 27\le {{x}_{i}} \le 45,\quad i=3,4,5. \end{cases} $$

### Example 8

(see [26])

$$ \textstyle\begin{cases} \min &x_{1}(-4x_{1}+x_{2}+2)-5x_{2}^{2} \\ \text{s.t.} & x_{1}-x_{2} \geq 0, \\ & (x_{1}+x_{2})(x_{1}-x_{2})\le 3, \\ & x_{1}x_{2}\le 2, \\ & 0 \le x_{1},x_{2}\leq 3. \end{cases} $$

### Example 9

(see [9, 27])

$$ \textstyle\begin{cases} \min &x_{1} \\ \text{s.t.} & x_{1}(1-x_{1})+x_{2}(8-x_{2})\le 16, \\ & x_{1}(x_{1}-6)+x_{2}(x_{2}-6)\le -14, \\ & 1 \le x_{1},x_{2} \leq 5.5. \end{cases} $$

### Example 10

(see Figs. 1–2)

$$ \textstyle\begin{cases} \min &(x_{1}-1)(x_{1}-2)(x_{2}-7)(x_{1}-5)-(x_{2}-1)(x_{2}-3)(x_{1}-4)^{2} \\ \textit{s.t.} & 0.1\le x_{1} \le 4.5, \quad\quad 0.1\le x_{2} \le 4.5. \end{cases} $$

**Figure 1:**
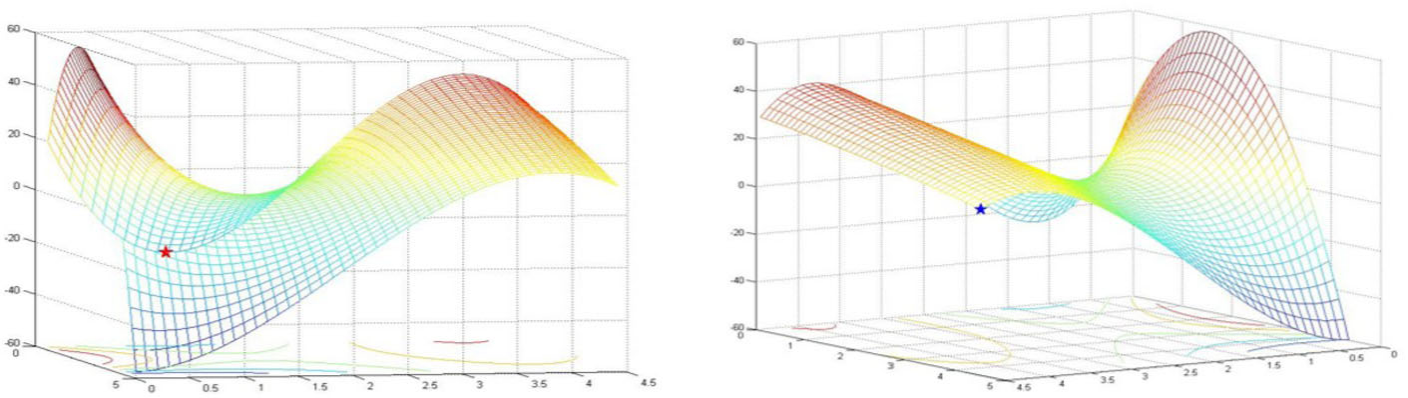
Two local solutions for Example 11 obtained by the proposed algorithm

**Figure 2:**
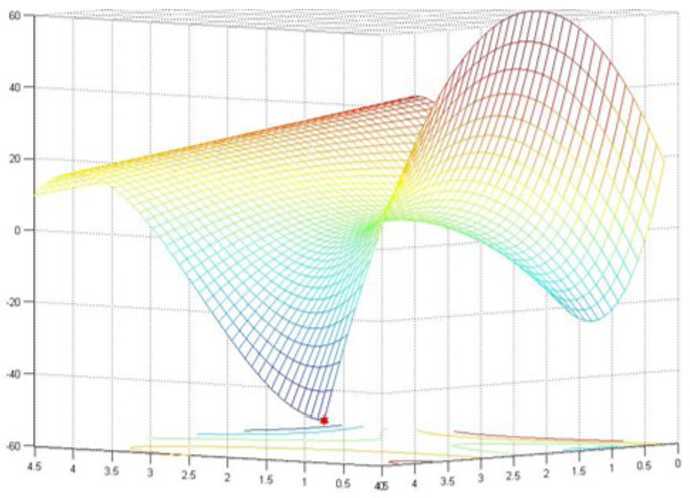
Global optimal solution for Example 11

When solving this example, by selecting $x^{0}=(0.1,0.4)$ and $y^{0}=(2,2)$ as starting points and applying the algorithm presented above, we obtained two different solutions $x_{\mathrm{opt}}=(4.5,4.5)$ and $y_{\mathrm{opt}}=(1.175957,0.1)$ with optimal objective values 9.625 and −24.641098, respectively. However, both of these two solutions are not the global optimal solution for Example 11. Actually, the only global optimal solution for Example 11 is $(0.1,4.5)$ with optimal value −58.905. Thus solutions $x_{\mathrm{opt}}=(4.5,4.5)$ and $y_{\mathrm{opt}}=(1.175957 0.1)$ just are two local solutions. The distribution of these three solutions for Example 11 are drawn in Figs. 1–2.

### Example 11

(st-qpk1)

$$ \textstyle\begin{cases} \min &2x_{1}-2x_{1}^{2}+2x_{1}x_{2}+3x_{2}-2x_{2}^{2} \\ \text{s.t.} & -x_{1}+x_{2} \le 1, \\ & x_{1}-x_{2}\le 1, \\ & -x_{1}+2x_{2} \le 3, \\ & 2x_{1}-x_{2} \le 3, \\ & 0 \le x_{1},x_{2}. \end{cases} $$

### Example 12

(ex8-1-7)

$$ \textstyle\begin{cases} \min &(x_{1}-1)^{2}+(x_{1}-x_{2})^{2}+(x_{2}-x_{3})^{3}+(x_{3}-x_{4})^{4}+(x _{4}-x_{5})^{4} \\ \text{s.t.} & x_{2}^{2}+x_{3}^{3}+x_{1}\le 6.24264068711929, \\ & -x_{3}^{2}-x_{2}^{2}-x_{1}\le -6.24264068711929, \\ & -x_{3}^{2}+x_{2}+x_{4}\le 0.82842712474629, \\ & x_{1}x_{5}=2, \\ & -5 \le x_{1},x_{2},x_{3},x_{4},x_{5} \leq 5. \end{cases} $$

### Example 13

(ex4-1-9)

$$ \textstyle\begin{cases} \min &-x_{1}-x_{2} \\ \text{s.t.} &8x^{3}_{1}-2x_{1}^{4}-8x_{1}^{2}+x_{2}\le 2, \\ &32x^{3}_{1}-4x_{1}^{4}-88x_{1}^{2}+96x_{1}+x_{2}\le 36, \\ & 0\le x_{1} \le 3, \quad\quad 0\le x_{2} \le 4. \end{cases} $$

### Example 14

(Small random test)

$$ \textstyle\begin{cases} \min &(c^{1}x+m_{1})^{\alpha _{1}}(c^{2}x+m_{2})^{\alpha _{2}}-(d^{1}x+r _{1})^{\beta _{1}}(d^{2}x+r_{2})^{\beta _{2}} \\ \text{s.t.} & (a^{1}x+s_{1})^{\gamma _{1}}(a^{2}x+s_{2})^{\gamma _{2}}-(b ^{1}x+t_{1})^{\theta _{1}}(b^{2}x+t_{2})^{\theta _{2}} \le 10+s_{1}^{ \gamma _{1}}s_{2}^{\gamma _{2}}-t_{1}^{\theta _{1}}t_{2}^{\theta _{2}}, \\ & 0 \le x \leq 1, \end{cases} $$

where $c^{1}$, $c^{2}$, $d^{1}$, $d^{2}$, $a^{1}$, $a^{2}$, $b^{1}$, $b^{2}$ are *n*-dimensional row vectors randomly generated in $[0,1]$, $m_{1}$, $m_{2}$, $r_{1}$, $r_{2}$, $s_{1}$, $s_{2}$, $t_{1}$, $t_{2}$ are all random real numbers between 0.001 and 1.001, $\alpha _{1}$, $\alpha _{2}$, $\beta _{1}$, $\beta _{2}$, $\gamma _{1}$, $\gamma _{2}$, $\theta _{1}$, $\theta _{2}$ are real numbers randomly generated in $[0,1]$, and we choose *n*-dimensional vector $(0.5,0.5,\ldots,0.5)$ as the starting point in each instance. The computational results of this problem are listed in Table 3.

Actually, the examples we chose in this section can be classified into four groups: Examples 1, 3, and 4 are taken from applications in optimal design; Examples 2, 5, 6, 7, 9 are numerical tests selected from some recent literature; Example 8 is computed to illustrate that the proposed algorithm can find just a local solution; and Example 14 is an example randomly generated with a relative large scale. Computational results are demonstrated in Tables 1–4 and Figs. 1–2. The computational results listed in the tables and figures show that our algorithm can perfectly solve problem (GLMP), and for most cases it can even attain a global optimal solution.

**Table 1:** Results of Examples 1–9 obtained by utilizing the presented method

Example	Start point	Iterations	Error in objective	Error constraint
1	(500,500,4200,500,400,340,300,600)	21	0	2.2204 × 10^−16^
2	(1.5,1.5)	5	0	8.8818 × 10^−16^
3	(100,100,100)	11	2.5070 × 10^−16^	0
4	(7,7,7,7,7,7,7,7)	44	0	0
5	(3,400)	15	0	0
6	(0.7,0.7,0.7,0.7)	8	0	0
7	(100,40,30,30,30)	5	0	2.2204 × 10^−16^
8	(1.5,1)	4	9.0949 × 10^−13^	0
9	(2,1.5)	6	3.5527 × 10^−15^	7.1054 × 10^−15^

**Table 2:** Results of the numerical comparison of Examples 5–9

Example	Methods	Optimal value	Optimal solution	CPU time
5	[9]	11.9541	(11.9604,0.8105,442.344)	0.416
	Ours	11.3497	(11.9604,0.681143,436.918047)	0.13252
6	[9]	−5.7416	(8.1244,0.6027,0.5660,5.6352)	42.3259
	Ours	−9.2952	(9.6867,0.5585,0.1000,5.3252)	0.8273
7	[27]	10,127.13	(78,32.999,29.995,45,36.7753)	1
	Ours	10,122.49325	(78,33,29.9957,45,36.775327)	0.331298
8	[26]	−15.0	(2,1)	120.580
	Ours	−15.0	(2,1)	0.3556
9	[9]	1.177081	(1.77091,2.17715)	0.2260
	[27]	1.1771243	(1.17712,2.17712)	0.26069
	Ours	1.177124	(1.177124,2.177124)	0.18726

**Table 3:** Results of numerical experiments (Examples 11–13)

Example	Best solution	Our solution	Best value	Our value
11 (st-qpk1)	–	(1,0)	–	0
12 (ex8-1-7)	(1.116635,1.220441,1.53779,1.97277,1.7911)	(1.116635,1.220441,1.53779,1.97277,1.7911)	0.0293	0.0291
13 (ex4-1-9)	(2.32952,3.1785)	(2.32952,3.1785)	−5.508	−5.511

**Table 4:** Computational results of random Example 14

Dimension	Iterations	CPU time	Error in objective	Error in constraint
*n* = 5	23	9.082938	0	4.4409 × 10^−16^
*n* = 10	20	14.92016	3.5527 × 10^−15^	2.6645 × 10^−15^
*n* = 20	17	36.85216	0	6.2172 × 10^−15^
*n* = 30	53	239.0432	3.5527 × 10^−15^	5.3291 × 10^−15^
*n* = 50	25	257.7263	0.7698 × 10^−15^	1.5395 × 10^−14^
*n* = 70	35	740.6696	8.8818 × 10^−16^	3.5527 × 10^−15^
*n* = 80	56	1583.152	1.7764 × 10^−15^	1.7764 × 10^−15^
*n* = 100	69	2043.238	0	3.1086 × 10^−15^

## Concluding remarks

In this paper, an inner approximation algorithm is presented for solving the generalized linear multiplicative programming problem. Local convergence property is proved and some numerical examples taken from application domain and recent literature are performed to verify the efficiency of the algorithm and quality of the solutions obtained. Results of the numerical tests show that this algorithm can effectively solve most generalized linear multiplicative problems to global optimality although it just has local convergence property.
